# The Application of Restriction Landmark Genome Scanning Method for Surveillance of Non-Mendelian Inheritance in F_1_ Hybrids

**DOI:** 10.1155/2009/245927

**Published:** 2010-01-27

**Authors:** Tomoko Takamiya, Saeko Hosobuchi, Tomotsugu Noguchi, Andrew H. Paterson, Hiroshi Iijima, Yasufumi Murakami, Hisato Okuizumi

**Affiliations:** ^1^Division of Genome and Biodiversity Research, National Institute of Agrobiological Sciences (NIAS), Tsukuba, Ibaraki 305-8602, Japan; ^2^Department of Biological Science & Technology, Faculty of Industrial Science & Technology, Tokyo University of Science, Noda, Chiba 278-8510, Japan; ^3^College of Pharmacy, Nihon University, Funabashi, Chiba 274-8555, Japan; ^4^Plant Genome Mapping Laboratory, University of Georgia, 162R C A G T, Athens, GA 30605, USA

## Abstract

We analyzed inheritance of DNA methylation in reciprocal F_1_ hybrids (subsp. *japonica* cv. Nipponbare × subsp. *indica* cv. Kasalath) of rice (*Oryza sativa* L.) using restriction landmark genome scanning (RLGS), and detected differing RLGS spots between the parents and reciprocal F_1_ hybrids. *MspI/Hpa*II restriction sites in the DNA from these different spots were suspected to be heterozygously methylated in the Nipponbare parent. These spots segregated in F_1_ plants, but did not segregate in selfed progeny of Nipponbare, showing non-Mendelian inheritance of the methylation status. As a result of RT-PCR and sequencing, a specific allele of the gene nearest to the methylated sites was expressed in reciprocal F_1_ plants, showing evidence of biased allelic expression. These results show the applicability of RLGS for scanning of non-Mendelian inheritance of DNA methylation and biased allelic expression.

## 1. Introduction

DNA methylation is very common in mammals and plants and plays an important role in the regulation of gene expression. For example, allele-specific DNA methylation regulates monoallelic expression, such as genomic imprinting [1–3], X-chromosome inactivation [4, 5], autosomal random monoallelic expression [6, 7], and allelic exclusion [8]. The methylation status in these phenomena is altered or inherited in a specific manner during development, growth, and reproduction. In mammals, DNA methylation patterns throughout the genome change dramatically during tumourigenesis [9], gametogenesis [10], or early development [11]. For example, imprinted genes are regulated by methylation of a differentially methylated region, and the allele-specific methylation pattern in the differentially methylated region is established in the germ cell line after erasing imprinting memory by demethylation [11]. In contrast, in plants, the methylation status of some genes is stably inherited through meiosis [12, 13]. Recent studies [14–16] have shown that methylation patterns can be altered in plant hybrids by introgression, and in allopolyploids. However, generational changes in methylation status and its inheritance in plants have remained unclear.

Restriction landmark genome scanning (RLGS) employs two-dimensional electrophoresis (2DE) of genomic DNA, which allows visualization of thousands of loci [17–20]. This method is appropriate for genome-wide methylation surveys [21–24]. We analyzed the inheritance of DNA methylation in the first filial generation (F1) hybrid between Oryza sativa L. subsp. japonica cv. Nipponbare and subsp. indica cv. Kasalath by RLGS, and detected altered inheritance and demethylation of specific RLGS spots in F1 plants [25]. In this study, we analyzed the appearance or disappearance of two altered spots in reciprocal F1 hybrids and selfed progeny, and detected an unexpected allelic expression bias.

## 2. Materials and Methods

### 2.1. Plant Materials and DNA Preparation

Seeds of *Oryza sativa* L. subsp. *japonica* cv. Nipponbare and subsp. *indica* cv. Kasalath were sown and grown in the field. Reciprocal hybrids were produced by crossing the same individual of each cultivar as the female parent on one culm and as the male parent on another culm. Crossing Nipponbare as the seed parent with Kasalath as the pollen parent gave F_1_ hybrids designated NKF_1_. The converse cross gave KNF_1_ hybrids. We grew plants of Nipponbare, Kasalath, NKF_1_ (nine individuals from the same parents), and KNF_1_ (nine individuals from the same parents), and the selfed progeny of the parents for 2 months, and then isolated the genomic DNA of each from the leaf blade and sheath by a standard CTAB extraction method [26].

### 2.2. RLGS and Identification of Target Spots

The methylation status of the parental Nipponbare and Kasalath, 9 NKF_1_ plants (NK1 to NK9), and 9 KNF_1_ plants (KN1 to KN9) was analyzed by an RLGS method with combinations of *Not*I–*Msp*I–*Bam*HI (hereafter [*Msp*I] pattern) or *Not*I-*Hpa*II-*Bam*HI ([*Hpa*II] pattern) restriction enzymes [22, 25]. Briefly, 0.4 *μ*g of genomic DNA was treated with 2 U DNA polymerase I (Nippon Gene, Tokyo, Japan) in 10 *μ*L of blocking buffer (10 mM Tris-HCl, pH 7.4, 10 mM MgCl_2_, 1 mM dithiothreitol (DTT), 0.4 *μ*M dGTP, 0.2 *μ*M dCTP, 0.4 *μ*M ddATP, and 0.4 *μ*M ddTTP) at 37°C for 20 minutes. Next, to inactivate DNA polymerase I, the sample was incubated at 65°C for 30 minutes. Thereafter, the genomic DNA was digested with 20 U *Not*I (NEB, Beverly, MA, USA) in a volume of 20 *μ*L, then the digested DNA was end-labeled by filling reaction with Sequenase ver. 2.0 (USB, Cleveland, OH, USA) in the presence of 0.33 *μ*M [*α*-^32^P] dGTP (3,000 Ci/mmol), 0.33 *μ*M [*α*-^32^P] dCTP (6,000 Ci/mmol), and 1.3 mM DTT at 37°C for 30 minutes. Thereafter, this reaction mixture was incubated at 65°C for 30 minutes to inactivate the enzyme. The sample was divided into two tubes. One was digested with 25 U *Msp*I (Toyobo, Tokyo, Japan), and the other was treated with 25 U *Hpa*II (Toyobo) at 37°C for 1 hour. Each sample was fractionated on an agarose disc gel (0.8% SeaKem GTG agarose, FMC Bioproducts, Rockland, ME, USA) in a 2.4 mm diameter × 63 cm long tube, and then electrophoresed in the 1st-dimensional (1-D) buffer (0.1 M Tris-acetate, pH 8.0, 40 mM sodium acetate, 3 mM EDTA, pH 8.0, 36 mM NaCl) at 100 V for 1 hour followed by 230 V for 23 hours. After 1-D electrophoresis, the gel was extruded from the tube and soaked for 30 minutes in the reaction buffer for *Bam*HI, and then the DNA in the gel was digested with 1500 U *Bam*HI for 2 hours. The gel was fused onto the top edge of a 50 cm (W) × 50 cm (H) × 0.1 cm (T) 5% vertical polyacrylamide gel using melted agarose (0.8%) to connect the gels. The 2nd-dimensional (2-D) electrophoresis parameters were Tris-borate-EDTA (TBE) buffer (50 mM Tris, 62 mM boric acid, 1 mM EDTA), at 100 V for 1 hour followed by 150 V for 23 hours. An area of 35 cm × 41 cm of the original gel was excised and dried. Autoradiography was performed for 3–10 days on film (XAR-5; Kodak, Rochester, NY, USA) at −80°C using an intensifying screen (Quanta III; Sigma-Aldrich, St. Louis, MO, USA), or for 1–3 days on an imaging plate (Fuji Photo Film, Tokyo, Japan). Finally, the imaging plate was analyzed by a BAS-2000 scanner (Fuji Photo Film). *Msp*I and *Hpa*II are restriction enzymes that recognize the same sequence, but have different methylation sensitivity. When the second C of the sequence CCGG is methylated (C^m^CGG), *Msp*I, but not *Hpa*II, cleaves the site. Conversely, neither *Msp*I nor *Hpa*II digests ^m^C^m^CGG or ^m^CCGG. Differences between [*Msp*I] and [*Hpa*II] patterns indicate a methylated CpG (C^m^CGG) at an *Msp*I/*Hpa*II site.

Target spots were identified using *in silico* RLGS computer software [22, 25], which simulates RLGS analysis of genome sequence data. The software calculates the length and mobility of each DNA fragment from the *Not*I to *Msp*I end or to the next *Not*I end in a 1D gel, and the DNA fragment length from the *Not*I to *Bam*HI end in a 2D gel to produce a 2D pattern (*in silico* RLGS pattern). We compared autoradiographic RLGS patterns with corresponding *in silico* RLGS patterns and identified each RLGS spot. The spots unidentified by *in silico *RLGS analysis were cloned and sequenced as previously described [22, 25] with specific cloning linkers: *Not*I linker (5′-GGCCGCATGAATGGCGCGCCAAAGA-3′, 3′-CGTACTTACCGCGCGGTTTCT-biotin-5′) and *Bam*HI linker (5′-GATCCTGTACTGCACCAGCAAATCC-3′, 3′-GACATGACGTGGTCGTTTAGG-5′).

### 2.3. Confirmation of Restriction Enzyme Sites by Digestion and PCR-Based DNA Methylation Analysis of Target Spots

 To compare methylation status among Nipponbare, Kasalath, and their F_1_s, we confirmed the presence of restriction enzyme sites in the parents. We designed flanking primers for the *Not*I and *Msp*I/*Hpa*II sites of each RLGS spot. Using 1 ng Nipponbare or Kasalath genomic DNA as a template, PCR was carried out with 0.4 U KOD plus polymerase (Toyobo,), 1.5 *μ*L flanking primers (10 pmol/*μ*L), 1 mM MgSO_4_, 0.2 mM dNTPs, and KOD buffer (total volume 20 *μ*L). PCR conditions were 94°C for 5 minutes followed by 30 cycles of 94°C for 15 s, 60°C for 30 s, and 68°C for 1 minutes. An aliquot of each PCR product was treated with *Not*I or *Msp*I. Then untreated and treated products were electrophoresed in an agarose gel (0.8%–3.0%), and the band sizes were compared to confirm that the sites were present and did not differ by any DNA size polymorphism. Next, we confirmed the methylation status of the *Not*I and *Msp*I/*Hpa*II sites of the RLGS spot. Genomic DNA (1 ng) of Nipponbare, Kasalath, or the reciprocal F_1_s was digested with 30 U *Not*I, *Msp*I, or *Hpa*II, and used as a PCR template. Undigested genomic DNA was used as a positive control. PCR was performed as described above.

### 2.4. Total RNA Isolation and Expression Analysis by RT-PCR

Using an RNeasy Plant Mini Kit (Qiagen, Tokyo, Japan), total RNA was isolated from the leaf blade and sheath of the same parents, NK5, NK7, KN5, and KN10. The 4 reciprocal F_1_ hybrids were chosen because two target RLGS spots (200 and 231) were detected in NK5 and KN5, but not in NK7 and KN10. First-strand cDNA was synthesized from 50 ng DNA-free samples with a ReverTra-Plus RT-PCR Kit (Toyobo, Osaka, Japan). The cDNA was used for RT-PCR analysis of each target gene. For spot 200, we used forward primer 5′-CACATCCTGATCACCGTCCA-3′ and reverse primer 5′-GTCCCAACCCGTGATCAAGTT-3′. For spot 231, we used forward primer 5′-ACTCAGGCTCAGATCGCCAT-3′ and reverse primer 5′-CCCGAGCTCCGTTTAGCATA-3′. Actin 1 was used as an internal standard (forward primer: 5′-TATGGTCAAGGCTGGGTTCG-3′, reverse primer: 5′-AACACAATACCTTGGGTACG-3′). PCR for each gene followed an initial denaturation for 2 minutes at 94°C, then 37 cycles of 10 s at 98°C, 30 s at 60°C, and 20 s at 68°C. The PCR products were analyzed by electrophoresis followed by ethidium bromide staining.

## 3. Results and Discussion

**Figure 1:**
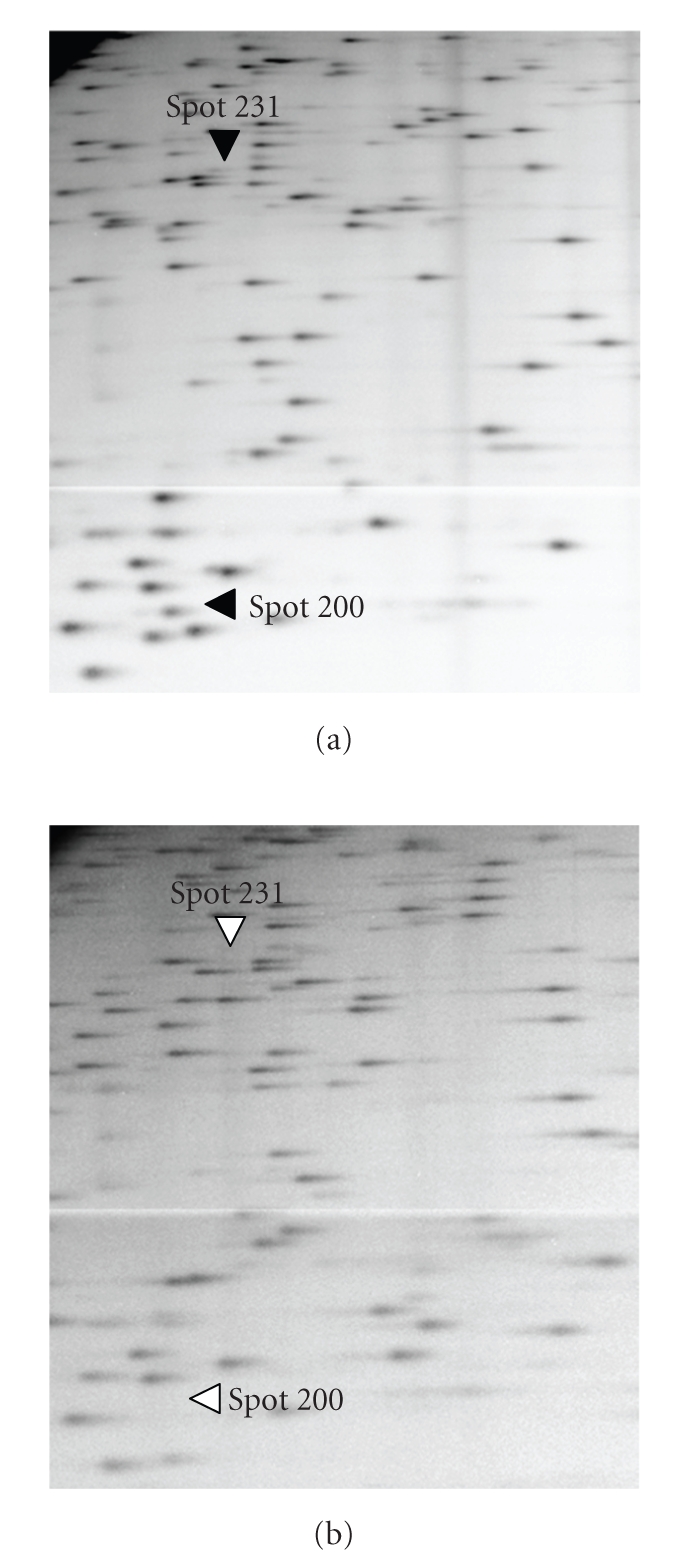
RLGS [*Msp*I] (*Not*I-*Msp*I-*Bam*HI) patterns of rice genomic DNA. Comparison of Nipponbare and Kasalath patterns revealed Nipponbare and Kasalath specific spots. (a) Nipponbare pattern. Spots 200 and 231 were detected at diminished spot intensities and are indicated by closed arrowheads. (b) Kasalath pattern. Neither spots 200 nor 231 were not detected.

**Figure 2:**
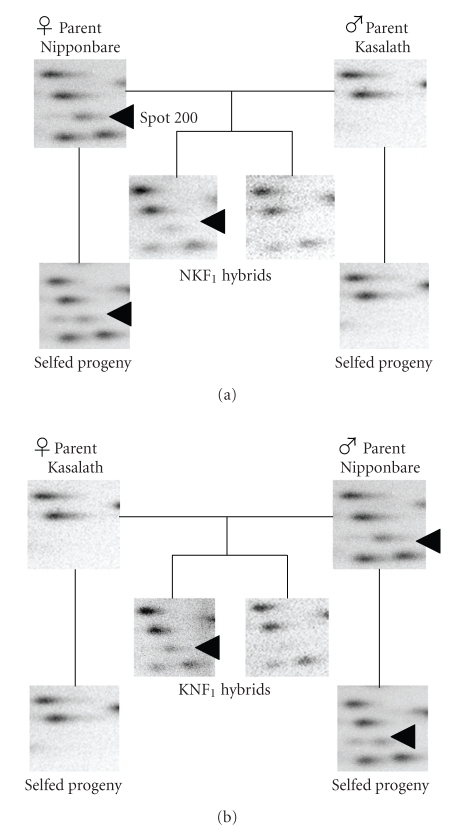
RLGS [*Msp*I] (*Not*I-*Msp*I-*Bam*HI) combination patterns of the parents, their selfed progeny, and their reciprocal F_1_ hybrids. Spot 200 (arrowhead) was detected in the [*Msp*I] patterns (Figure 1) and [*Hpa*II] (*Not*I-*Hpa*II-*Bam*HI) patterns (data not shown) of Nipponbare and its selfed progeny. The presence or absence of the spot segregated in both F_1_ populations (NKF_1_ and KNF_1_). The spot intensity of this spot was half that of the others.

**Figure 3:**
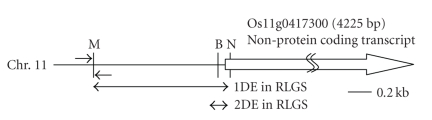
Location of spot 200. Schematic of the region of chromosome 11 containing the restriction enzyme sites located in the region 5′ to the transcription start site of the non-protein coding transcript (Os11g0417300). The DNA fragments were digested at the *Not*I (N) and *Msp*I/*Hpa*II (M) sites and fractionated by one dimensional electrophoresis. Next, the DNA fragments that were digested at the *Bam*HI (B) sites were fractionated by two dimensional electrophoresis, which allowed detection of the B-N fragment as an RLGS spot. Spot 200 corresponds to the fragment between the N and B sites. The N and M sites were identified in the parental Nipponbare and Kasalath, and in both reciprocal hybrids. The B site was only absent in Kasalath, resulting in the absence of spot 200 in the RLGS pattern.

Analysis of the RLGS patterns of the parents and the reciprocal hybrids showed variations in some spots between samples, reflecting changes in DNA methylation. One such altered spot was spot 200, which was detected in both the [*Msp*I] and [*Hpa*II] patterns of Nipponbare at a diminished spot intensity (half the intensity of the surrounding spots), but was absent in Kasalath (Figures 1 and 2). Cloning and sequencing of this DNA fragment placed it in the 5′ region of a non-protein coding transcript (Os11g0417300) (Figure 3). Comparison of the relative spot positions between autoradiographic RLGS patterns of the parental Nipponbare and *in silico* RLGS pattern derived from Nipponbare genome sequence data revealed that the DNA fragments digested at the *Not*I (N) and *Msp*I (M) sites were fractionated by 1-D electrophoresis, and the DNA fragments digested at the N and *Bam*HI (B) sites were fractionated by 2-D electrophoresis as spot 200 (Figure 3). By restriction enzyme digestion and sequencing, we confirmed the existence of N, M, and B in the parental Nipponbare (data not shown). In the parental Kasalath, there were N and M sites, but no B site (data not shown). The results of RLGS analysis of the NKF_1_ and KNF_1_ hybrids showed that the presence or absence of spot 200 segregated 1:1 in both populations (Figure 2 and Table 1). The diminished spot intensity in the parental Nipponbare and its segregation in F_1_ hybrids imply that the *Msp*I/*Hpa*II site of spot 200 is methylated heterozygously in Nipponbare. Accordingly, it was assumed that spot 200 was detected in the F_1_ individuals that had a non-methylated M site, and not detected in the F_1_ individuals that had a methylated M site. Additionally, spot 200 was detected in all selfed progeny (nine individuals) of Nipponbare at half intensity (Figure 2 and Table 1). In RLGS analysis, halved intensity of a spot indicates a heterozygote, which was confirmed theoretically and practically in earlier studies [27, 28]. From this observation, it was assumed that the M site was methylated heterozygously in the selfed progeny as well as the parental Nipponbare because of non-Mendelian inheritance of methylation.

**Figure 4:**
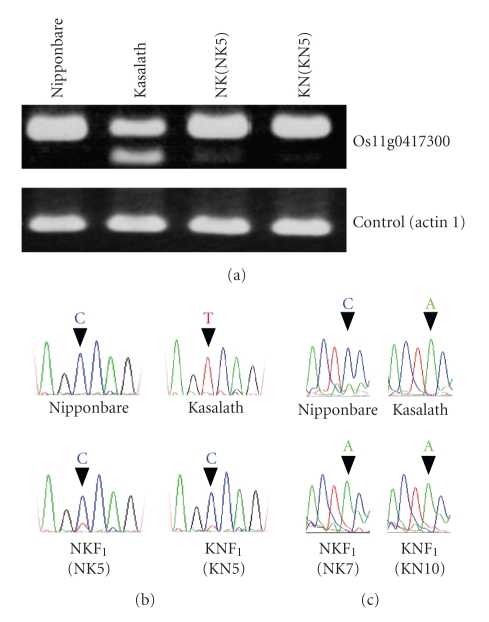
Expression analysis of the nearest gene to the methylated site. (a) RT-PCR showed that a non-protein coding transcript (Os11g0417300) was expressed in leaf blade and sheath of Nipponbare, Kasalath, NKF_1_, and KNF_1_ plants. (b) Sequence analysis of the RT-PCR products of the expressed Os11g0417300 allele. The single nucleotide polymorphism between Nipponbare (C) and Kasalath (T) is indicated in the RT-PCR products by arrowheads. Specific expression of the Nipponbare allele was confirmed by detection of base C in both NK5 and KN5 plants. (c) Sequence analysis of RT-PCR products of the expressed Os01g0327900 allele. The single nucleotide polymorphism in RT-PCR products between Nipponbare (C) and Kasalath (A) is indicated by arrowheads. Specific expression of the Kasalath allele was confirmed by detection of base A in both NK7 and KN10 plants.

We suspected that the methylation status correlated with expression of the nearest gene. Therefore, we analyzed the expression of the non-protein coding transcript (Os11g0417300) that is the nearest gene to the *Msp*I/*Hpa*II site of spot 200 (Figure 3). The cDNA (GenBank accession No. **AK109537**) of the non-protein coding transcript, which was previously isolated, is expressed in flower, leaf, and panicle (http://www.ncbi.nlm.nih.gov/sites/entrez?db=unigene& cmd=search&term=AK109537). We analyzed expression of the gene by RT-PCR. Total RNA was isolated from the leaf blade and sheath of the parental Nipponbare, parental Kasalath, two NKF_1_ individuals (NK5 and NK7), and two KNF_1_ individuals (KN5 and KN10). Spot 200 was detected in the patterns of NK5 and KN5, but not in the patterns of NK7 and KN10. The cDNAs were PCR-amplified and separated by agarose gel electrophoresis (Figure 4(a)). The non-protein coding transcript was expressed in the leaf blade and sheath of the parents, NKF_1_s, and KNF_1_s (data for NK7 and KN10 are not shown but gave the same result). Next, we sequenced the RT-PCR products to reveal the parental origin of the expressed sequence in the F_1_ hybrids. The presence of a single nucleotide polymorphism (C/T) between Nipponbare and Kasalath allowed this distinction to be made. Sequence analysis of the RT-PCR products from NK5 and KN5, which had spot 200 in their RLGS patterns, showed allelic expression bias for the Nipponbare allele (Figure 4(b)); analysis of NK7 and KN10, which did not have spot 200, also showed bias (data not shown but gave the same result). The bias in the reciprocal hybrids was strong, and implied monoallelic expression of the Nipponbare allele. In addition, we detected a Kasalath-specific splicing variant as a smaller transcript with an expression level lower than that of the Nipponbare allele. This transcript was absent in NKF_1_ and KNF_1_. Sequencing this transcript revealed a splicing variant that leads to a 76-bp deletion at the 3′ end of exon 2.

The non-Mendelian spot 231 showed the same behavior as spot 200 on RLGS. The spot intensity was half that of the surrounding spots and the presence or absence of this spot also segregated 1 : 1 in NKF_1_ and KNF_1_. Additionally, spot 231, like spot 200, was detected in all selfed progeny of Nipponbare. We similarly analyzed the expression of the nearest gene (DUF295 family protein Os01g0327900) in two NKF_1_ (NK5 and NK7) and two KNF_1_ (KN5 and KN10) individuals. Sequence analysis of the RT-PCR products showed that only the Kasalath allele was expressed in NK5, NK7, KN5, and KN10 (Figure 4(c) shows the results for NK7 and KN10; data for NK5 and KN5 are not shown but gave the same result). In this study, we have given two examples of the nearest gene to a heterozygous methylated site showing allelic expression bias. 

Recently, monoallelic expression in F_1_ hybrids of plants has been reported. Zhuang and Adams [29] reported that in *Populus* interspecific hybrids, 17 out of 30 genes analyzed showed >1.5-fold expression bias for one of two alleles, with monoallelic expression of one gene [29], while intraspecific maize hybrids have shown unequal expression of parental alleles [30–32]. Therefore, histone modification or DNA methylation is considered one cause of allelic expression bias.

Elucidation of the significance and mechanism of regulation of monoallelic expression requires detection of more RLGS spots showing non-Mendelian inheritance along with the analysis of the methylation status of the corresponding DNA sequence and the expressed allele. Further expression analyses of genes in F_1_s having different genetic backgrounds will support our findings for application to other genes. Moreover, revealing the function of the splicing variant of Kasalath in F_1_ hybrids may provide better understanding of the mechanism of allelic exclusion inducing heterosis, hybrid weakness, and genome barriers.

## 4. Conclusion

Our findings clearly demonstrate that the RLGS method can be successfully applied to survey non-Mendelian inheritance of DNA methylation. Consequently, we detected two loci showing non-Mendelian inheritance and allelic expression bias in F_1_ hybrids of rice. The systematic scanning has the following advantages: (1) easy detection of candidates for non-Mendelian inheritance of DNA methylation by simple comparison of spot patterns between parents and F_1_ hybrids, (2) low cost and quick yield results in only 3 days, and (3) detection of potentially more non-Mendelian spot candidates using different restriction enzyme combinations in RLGS.

## Figures and Tables

**Table 1:** Summary of RLGS pattern of spot 200.

Generation		RLGS pattern of spot 200	
		*Msp*I patterns (intensity)	*Hpa*II patterns (intensity)
Parent	Nipponbare	Present (1/2)	Present (1/2)
Parent	Kasalath	Absent	Absent
Selfed progeny (9 individuals)	Nipponbare	Present (1/2)	Present (1/2)
Selfed progeny (4 individuals)	Kasalath	Absent	Absent
NKF_1_ (9 individuals)	Nipponbare × Kasalath	Segregated5 present: 4 absent (1/2: 0)	Segregated4 present: 5 absent (1/2: 0)
KNF_1_ (9 individuals)	Kasalath × Nipponbare	Segregated7 present: 2 absent (1/2: 0)	Segregated4 present: 5 absent (1/2: 0)

## References

[1] Tiwari S, Schulz R, Ikeda Y (2008). MATERNALLY EXPRESSED PAB C-TERMINAL, a novel imprinted gene in Arabidopsis, encodes the conserved C-terminal domain of polyadenylate binding proteins. *The Plant Cell*.

[2] Nowack MK, Shirzadi R, Dissmeyer N (2007). Bypassing genomic imprinting allows seed development. *Nature*.

[3] Daura-Oller E, Cabre M, Montero MA, Paternain JL, Romeu A (2009). A first-stage approximation to identify new imprinted genes through sequence analysis of its coding regions. *Comparative and Functional Genomics*.

[4] Avner P, Heard E (2001). X-chromosome inactivation: counting, choice and initiation. *Nature Reviews Genetics*.

[5] Shen Y, Matsuno Y, Fouse SD (2008). X-inactivation in female human embryonic stem cells is in a nonrandom pattern and prone to epigenetic alterations. *Proceedings of the National Academy of Sciences of the United States of America*.

[6] Sano Y, Shimada T, Nakashima H (2001). Random monoallelic expression of three genes clustered within 60 kb of mouse t complex genomic DNA. *Genome Research*.

[7] Gimelbrant A, Hutchinson JN, Thompson BR, Chess A (2007). Widespread monoallelic expression on human autosomes. *Science*.

[8] Fraenkel S, Mostoslavsky R, Novobrantseva TI (2007). Allelic ‘choice’ governs somatic hypermutation in vivo at the immunoglobulin *κ*-chain locus. *Nature Immunology*.

[9] Zeschnigk M, Tschentscher F, Lich C, Brandt B, Horsthemke B, Lohmann DR (2003). Methylation analysis of several tumour suppressor genes shows a low frequency of methylation of CDKN2A and RARB in uveal melanomas. *Comparative and Functional Genomics*.

[10] Monk M, Boubelik M, Lehnert S (1987). Temporal and regional changes in DNA methylation in the embryonic, extraembryonic and germ cell lineages during mouse embryo development. *Development*.

[11] Tada S, Tada T, Lefebvre L, Barton SC, Surani MA (1997). Embryonic germ cells induce epigenetic reprogramming of somatic nucleus in hybrid cells. *The EMBO Journal*.

[12] Jacobsen SE, Meyerowitz EM (1997). Hypermethylated SUPERMAN epigenetic alleles in Arabidopsis. *Science*.

[13] Kakutani T, Munakata K, Richards EJ, Hirochika H (1999). Meiotically and mitotically stable inheritance of DNA hypomethylation induced by ddm1 mutation of *Arabidopsis thaliana*. *Genetics*.

[14] Xu Y, Zhong L, Wu X, Fang X, Wang J (2009). Rapid alterations of gene expression and cytosine methylation in newly synthesized *Brassica napus* allopolyploids. *Planta*.

[15] Liu Z, Wang Y, Shen Y, Guo W, Hao S, Liu B (2004). Extensive alterations in DNA methylation and transcription in rice caused by introgression from *Zizania latifolia*. *Plant Molecular Biology*.

[16] Woo HR, Richards EJ (2008). Natural variation in DNA methylation in ribosomal RNA genes of *Arabidopsis thaliana*. *BMC Plant Biology*.

[17] Hayashizaki Y, Hirotsune S, Okazaki Y (1993). Restriction landmark genomic scanning method and its various applications. *Electrophoresis*.

[18] Yoshikawa H, Nagai H, Matsubara K, Fujiyama A (1993). Two-dimensional gel electrophoretograms of human chromosome specific restriction DNA fragments. *Biochemical and Biophysical Research Communications*.

[19] Okamoto H, Takamiya T, Saito A (2006). Development of a new cultivar-discrimination method based on DNA polymorphism in a vegetatively propagated crop. *Japan Agricultural Research Quarterly*.

[20] Ichida H, Maeda K, Ichise H (2007). In silico restriction landmark genome scanning analysis of Xanthomonas oryzae pathovar oryzae MAFF 311018. *Biochemical and Biophysical Research Communications*.

[21] Hayashizaki Y, Shibata H, Hirotsune S (1994). Identification of an imprinted U2af binding protein related sequence on mouse chromosome 11 using the RLGS method. *Nature Genetics*.

[22] Takamiya T, Hosobuchi S, Asai K (2006). Restriction landmark genome scanning method using isoschizomers (Mspl/Hpall) for DNA methylation analysis. *Electrophoresis*.

[23] Takamiya T, Ohtake Y, Hosobuchi S (2008). Application of RLGS method for detection of alteration in tissue cultured plants. *Japan Agricultural Research Quarterly*.

[24] Costello JF, Hong C, Plass C, Smiraglia DJ (2009). Restriction landmark genomic scanning: analysis of CpG islands in genomes by 2D gel electrophoresis. *Methods in Molecular Biology*.

[25] Takamiya T, Hosobuchi S, Noguchi T (2008). Inheritance and alteration of genome methylation in F_1_ hybrid rice. *Electrophoresis*.

[26] Murray MG, Thompson WF (1980). Rapid isolation of high molecular weight plant DNA. *Nucleic Acids Research*.

[27] Hayashizaki Y, Hirotsune S, Okazaki Y (1994). A genetic linkage map of the mouse using restriction landmark genomic scanning (RLGS). *Genetics*.

[28] Okuizumi H, Okazaki Y, Hayashizaki Y, Hayashizaki Y, Watanabe S (1997). RLGS spot mapping method. *Restriction Landmark Genomic Scanning (RLGS)*.

[29] Zhuang Y, Adams KL (2007). Extensive allelic variation in gene expression in populus F_1_ hybrids. *Genetics*.

[30] Guo M, Rupe MA, Zinselmeier C, Habben J, Bowen BA, Smith OS (2004). Allelic variation of gene expression in maize hybrids. *The Plant Cell*.

[31] Springer NM, Stupar RM (2007). Allelic variation and heterosis in maize: how do two halves make more than a whole?. *Genome Research*.

[32] Springer NM, Stupar RM (2007). Allele-specific expression patterns reveal biases and embryo-specific parent-of-origin effects in hybrid maize. *The Plant Cell*.

